# Development of integrated care pathways: toward a care management system to meet the needs of frail and disabled community-dwelling older people

**DOI:** 10.5334/ijic.976

**Published:** 2013-05-17

**Authors:** Nicole Dubuc, Lucie Bonin, André Tourigny, Luc Mathieu, Yves Couturier, Michel Tousignant, Cinthia Corbin, Nathalie Delli-Colli, Michel Raîche

**Affiliations:** Research Centre on Aging, University Institute of Geriatrics of Sherbrooke, and Faculty of Medicine and Health Sciences, Université de Sherbrooke, Sherbrooke, Québec, Canada; Agence de la santé et des services sociaux de la Mauricie-Centre-du-Québec, Trois-Rivières, Québec, Canada; Institut national de santé publique du Québec, Centre de recherche du Centre hospitalier affilié, Québec, Canada; Faculty of Medicine and Health Sciences, Université de Sherbrooke, Sherbrooke, Québec, Canada; Research Centre on Aging, University Institute of Geriatrics of Sherbrooke, and Faculty of Arts, Humanities and Social Sciences, Université de Sherbrooke, Sherbrooke, Québec, Canada; Research Centre on Aging, University Institute of Geriatrics of Sherbrooke, and Faculty of Medicine and Health Sciences, Université de Sherbrooke, Sherbrooke, Québec, Canada; Research Centre on Aging, University Institute of Geriatrics of Sherbrooke, Sherbrooke, Québec, Canada; Research Centre on Aging, University Institute of Geriatrics of Sherbrooke, and Faculty of Arts, Humanities and Social Sciences, Université de Sherbrooke, Sherbrooke, Québec, Canada; Research Centre on Aging, University Institute of Geriatrics of Sherbrooke, Sherbrooke, Québec, Canada

**Keywords:** integrated care pathways, older people, disability, frailty, home care, client-centered

## Abstract

**Introduction:**

The home care and services provided to older adults with the same needs are often inadequate and highly varied. Integrated care pathways (ICPs) can resolve these issues. The aim of this study was to develop the content of ICPs to follow-up frail and disabled community-dwelling older people.

**Theory and method:**

A rigorous process was applied according to a series of steps: identification of desirable characteristics and a theoretical framework; review of evidence-based practices and current practices; and determination of ICPs by an interdisciplinary task team.

**Results:**

ICPs are intended to prevent specific problems, maximize independence, and promote successful aging. They are organized according to a dynamic process: (1) needs assessment and assessment of risk/protection factors; (2) data-collection summary and goals identification; (3) planning of interventions from a client-centered view; (4) coordination, delivery, and follow-up; and (5) identification of variances, as well as review and adjustment of plans.

**Conclusion:**

Once computerized, these ICPs will facilitate the exchange of information as well as the clinical decision-making process with a perspective to adequately matching the needs of an individual person with resources that delay or slow the progression of frailty and disability. Once aggregated, the data will also support managers in organizing teamwork and follow-up for clients.

## Introduction

Canada faces accelerated aging of its population and Quebec, the country's second most populous province, is no exception. In Quebec, people aged over 65 constitute 16% of the population and they will represent 26% by 2031 [1]. Even though seniors may consider themselves to be in good health, they form an extremely heterogeneous group. They may display different chronic illnesses and comorbidities, various levels and profiles of disability, and present some components of frailty such as sarcopenia or poor endurance [2]. As most older adults want to live at home surrounded by family and friends, they are increasingly seeking support and health-related services from home and community-based services [3, 4]. Given the aging population and the benefits of providing care and services in the community, the home-care sector will assume greater importance in the future [5, 6].

In our province, health- and social-services centers (HSSCs) provide the population of a local territory with a wide range of primary-care services, including public-health services like home care, in collaboration with other partners such as community and private organizations. They are also responsible for establishing referral and follow-up mechanisms to ensure access to secondary and tertiary care (specialized and highly specialized services) [7]. Considering the variety of providers and resources that may be involved, however, continuity-related problems were frequently observed in the past [8]. Accordingly, integrated service networks (ISNs) have gradually been established and have been shown to improve service efficacy [9–11]. Over the past ten years, a number of ISN projects for frail elderly clients have been conducted and even motivated the reform of the province’s health network begun in 2003 [7]. Despite many improvements in the organization of health-care systems, however, the heterogeneity of the elderly population contributes to the clinical challenges that professionals face in providing care [12]. Research carried out over the past few years has shown that provision and quality of home care for individuals with the same needs varies considerably [6, 13–15]. These variations lead to inequities in access, especially for those with severe disabilities [16]. Our home-care services show the same situation, while facing a demand that exceeds their current capacity [17, 18]. Therefore, management’s goal is to establish more efficient practice and service-organization models for use in our ISNs. The goal is to favor equitable access to disabled elders with similar needs, to include support and prevention services, and to promote independence in daily life for older persons living in the community.

Integrated care pathways (ICPs) have been promoted internationally [19–26] as a response to concerns for patient safety, variability in care, and increasing care costs. ICPs usually define best practices or essential care components for a group of persons with a given diagnosis or health condition and they determine locally-agreed-upon, multidisciplinary practices [27]. ICPs are particularly useful in identifying care variations or gaps when the services provided do not match some aspects of the established standardized pathway. Thus, remedial action can then be taken and the information may serve as quality control or as a review device [21, 28]. Positive clinical effects of integrated care pathways have been observed for different clinical situations. For example, studies have shown that ICPs lower hospital readmissions and length of stay, while leading to improvement in service quality, safety, and efficiency [22, 27–31]. They also improve documentation and communication with clients and enhance consistency and continuity of care [31, 32]. In home care, ICPs may be a good strategy that offers ways to achieve better integration among practitioners, community-based services, and other health- and social-care service providers. Until now, however, ICPs have mainly been limited to monitoring older people with a specific medical diagnosis (e.g. diabetes or pulmonary disease) [19, 22, 33, 34]. To date, no ICP has been available for following up older adults with different disability profiles, evidencing certain components of frailty, living in the community, and receiving long-term care services.

## Objectives

The goal of this study was to develop the content of electronic integrated care pathways (ICPs) that will ensure the follow-up of frail and disabled older adults living in the community.

## Theory

### Defining disability and frailty

Disability is defined as experiencing difficulty in performing activities in any area of life in which the nature of role-task behavior varies from fairly basic self-care activities to advanced and complex social, work, and leisure activities [35]. Disability can be of different degrees (mild to severe) and types (e.g. cognitive decline or mobility problems), as they result from a complex interaction between a person's features and that of the environment in which he or she lives [2, 35–39]. The measurement of disability is considered essential in geriatric assessment and is used as a basis to establish eligibility for long-term services in many countries [40–42]. It is also a dynamic process characterized by frequent transitions between states of independence and disability or disability profiles [43, 44]. In the last decade, many complex interventions were designed to prevent, restore, or maintain independence in older persons and have shown promising results [45–49].

More recently, with a view to describing other aspects of the health status of the elderly, many researchers have taken an interest in the concept of frailty. Although this concept still remains difficult to define, researchers explain it as a biological syndrome involving reduced physiological reserves and resistance to stressors resulting from the cumulative loss of multiple physiological systems that affect the individual’s ability to maintain an equilibrium with his/her environment, or to reestablish that equilibrium subsequent to disruptive events [50, 51]. Frail seniors thus also form an extremely heterogeneous clientele [50, 52]; they may display such diverse symptoms as weight loss or sarcopenia, low activity level, poor endurance, or weakness. The most recent and common components of frailty identified are physical functioning, gait speed, and cognition [53]. Disability investigated as an outcome measure of frailty has also frequently cropped up in recent studies. Following the example of disability, frailty seems to be recognized as a dynamic rather than static condition [53].

### Defining integrated care pathways

ICP has many synonyms (e.g. care pathways, critical pathways, and clinical pathways) and ICP core features can vary widely [23, 24, 54, 55]. ICPs first appeared in the 1970s, but their development accelerated during the 1990s, as concerns about cost management and quality of care increased [56]. They were generally aimed at short-term-care clientele requiring huge service volumes or expensive procedures, or who were at high risk of complications [56]. Several ICP models can now be found around the world [22, 24, 25, 27]. While ICPs take into account the activities of all members of the interdisciplinary team and reflect best clinical practices, they must also be realistically designed and reflect local and regional issues [26]. For the purpose of this study, we have based our work on the definition developed by the European Pathway Association [57]. They define integrated care pathway as “a complex intervention for the mutual decision making and organization of predictable care for a well-defined group of patients during a well-defined period. Defining characteristics of pathways include: an explicit statement of the goals and key elements of care based on evidence, best practice and patient expectations; the facilitations of the communication and coordination of roles, and sequencing the activities of the multidisciplinary care team, patients and their relatives; the documentation, monitoring, and evaluation of variances and outcomes; and the identification of relevant resources” [27].

## Method

**Figure 1:**
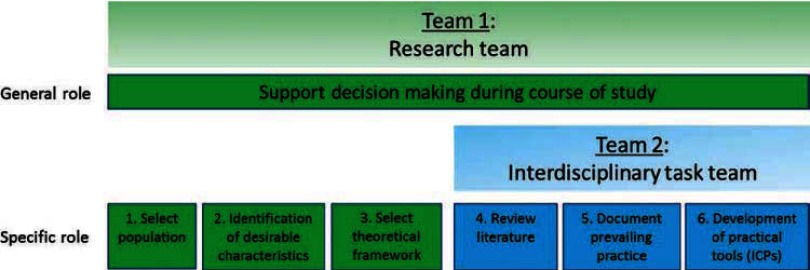
Overview of teams and roles in methodology.

ICPs are generally developed according to a series of steps. In this study, the work of De Luc et al. [58–60], as well as that of the European Pathway Association [57], partially guided the steps taken to develop the content of these ICPs. Two teams cooperated iteratively during their development. A research team, responsible for decision making during the course of this study, selected the target population, defined the desirable characteristics, and selected a theoretical framework (see Figure 1). An interdisciplinary task team conducted literature reviews, documented prevailing practices, and developed practical tools. Members of this task team had expertise in research and clinical experience in gerontology and in home care. The team comprised two nurses, a psychologist, two occupational therapists, a physiotherapist, a social worker, and two members of the research team (a nurse and a physician). Representatives of other disciplines, such as a dietician, pharmacist, and speech therapist, were consulted as necessary.

### Population selection

In this study, ICPs were designed to address the needs of older adult clients with frailty and disability living in community-based integrated care network. More precisely, they were designed for persons with various disability profiles as defined by the 14 Iso-SMAF classification profiles [38]. Each Iso-SMAF profile (‘iso’ means ‘homogeneous’) is defined with specific characteristics according to the functional autonomy measurement system (SMAF) [61]. The Iso-SMAF classification and the SMAF tool have been assessed for validity and reliability in previous studies [38, 42, 61, 62]. In this classification, there is a progression of disabilities from the first to the fourteenth profiles. To summarize, they can be grouped into four broad categories: persons with disabilities mainly in instrumental activities of daily living (IADLs) (profiles 1, 2, and 3); persons with IADL and ADL disabilities with predominant mobility problems (profiles 4, 6, and 9); persons with IADL and ADL disabilities with predominant cognitive problems (profiles 5, 7, 8, and 10); and persons with mixed and severe disabilities (profiles 11, 12, 13, and 14) [38]. The profile is automatically generated from the SMAF [61] which evaluates 29 functions, covering five domains of activity: ADLs (e.g. eating, washing, dressing, continence), mobility (e.g. transfers, using the stairs, walking indoors), communication (vision, hearing, talking), mental functions (e.g. memory, orientation), and IADLs (e.g. housekeeping, preparing meals, managing medication). In the province of Quebec, the SMAF has been included since 2001 in the Multiclientele Assessment Tool (named OEMC, French acronym for “*Outil d’évaluation multiclientèle*”), which has been approved by the government for use in all long-term-care facilities, including home health-care agencies [63]. Thus, the development of our ICPs was linked to the OEMC.

### Identification of ICP characteristics desirable in a Quebec context

ICP development, which is an extension of the work conducted by the PRISMA (Program on Research for Integrating Services for the Maintenance of Autonomy) [8, 10] and SIPA (System of Integrated Services for Older Persons) [11] research teams on care and service integration, was aimed at facilitating the continuity and coordination of care and services for community-dwelling elderly persons. These research teams are now linked under the CIHR (Canadian Institute of Health Research) Team in frailty and aging.

From the viewpoint of the client, continuity consists of care that is consistent and connected in time. From the viewpoint of the service provider, it consists in collecting enough data to be able to put his/her professional skills to best possible use [64]. If care is to be properly coordinated, efforts at all levels must be united and synchronized, so as to enable the organization to reach its objectives. This requires meaningful, effective communication among service providers. In monitoring community-dwelling older people, our ICPs have been developed to help professionals and managers organize services efficiently, thereby supporting older people at home, as well as optimizing their functioning in all spheres of life. Interventions are aimed at health promotion and disability prevention, as well as at the care and services needed for elders and their families, with particular emphasis on restorative or re-ablement care [12, 47, 48]. Lastly, ICPs attempt to integrate a global approach that is centered on the needs of the client and his/her caregivers, so as to support personalized-care delivery [27, 65].

Certain basic characteristics were deemed important. At the very least*, at the professional level,* it is expected that ICPs will (i) be developed in accordance with a logical, dynamic intervention process based on evidence-based practice; (ii) for the most part, use common, standardized terminology; (iii) assist in planning interventions with the expectations of the individual and family caregivers; (iv) assist in clinical decision-making and the interdisciplinary and inter-service exchange of information (continuity and coordination); and (v) include a list of care and services that can be carried out by public, private, and community resources working together. *At the organizational level,* ICPs will (i) constitute joint tools to support the work of several different teams or services and (ii) assist in identifying the anticipated contribution of each partner on the service continuum.

Lastly, ICPs will be incorporated into a user-friendly, highly effective computerized system that will provide clinical teams with feedback via real-time data processing. This will also prove useful for managers in supporting the organization of work teams, organization of services, and monitoring clienteles. Studies have shown that using a computerized system in the health-care sector generates better data cohesion and accuracy, makes record-keeping standards easier to reach, and prevents data-entry duplication. We have noted, however, that computer applications appear more often in hospitals than elsewhere, such as long-term care, clinics, and the home-care sector. Although several applications are interdisciplinary oriented, they are often used within the same organization instead of between institutions.

Many authors agree that, in the case of ICPs, in particular, information systems are a key component of the infrastructure required to support implementation. Matthews and collaborators [66] underline that, the more the information entry is performed at source, the greater the chances that it will be used to make the required changes in clinical practice. Although a significant proportion of ICPs are currently available in paper version, Chu [67] says that this format would significantly limit their use. Computerization would have numerous advantages: (i) promoting data collection of better quality; (ii) facilitating online access to clinical practice guides or technical information; (iii) allowing data assembly (e.g. creation of lists of medication or services); (iv) speeding up record review; (v) adding or removing certain elements to create a plan adapted to a client’s particular needs; (vi) enabling easy access to client records by different professionals from different locations; and (vii) obtaining an overview of a client’s record according to the discipline concerned [60].

While computerized systems have numerous advantages, several factors can facilitate or limit their use and success. These factors are clientele characteristics (older people and caregivers), users (professionals and managers) and the computerized environment in the target organization, work organization (e.g. ways of making interpersonal relations, consent, confidentiality, system performance), and the impacts on patients and professionals. These factors must be taken into consideration when assessing computerized applications.

### Selection of a theoretical framework

**Figure 2:**
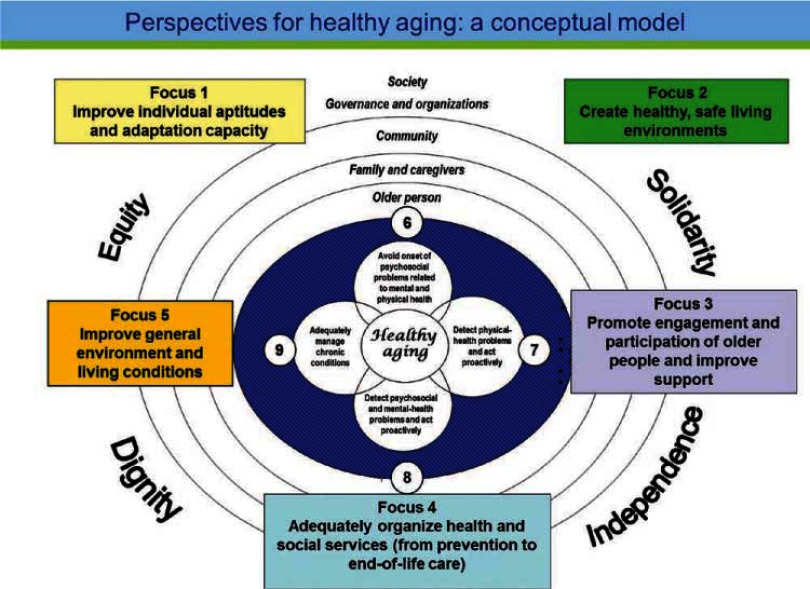
Healthy aging conceptual model. By Cardinal and collaborators [68].

ICP design was based on the ‘healthy aging’ model developed by Institut national de santé publique du Québec (Quebec’s Provincial Public-Health Institute) [68]. This framework, which targets healthy seniors as well as those with disabilities or chronic illnesses, takes account of all the intervention factors and strategies intended to maintain or improve the health of older person. The proposed model includes nine main focuses of intervention. The first five involve action on the main health determinants; the other four, on risk factors and health conditions through the prevention of specific problems and the optimization of remaining abilities in the population concerned (see Figure 2). For the purposes of this study, we have concentrated on eight out of the nine focuses:

- Improve the individual abilities and adaptability of elderly clients; support self care (Focus 1).
- Create healthy, safe, and secure living environments (home and immediate environment) (Focus 2).
- Promote client engagement and participation in society, and improve the support available for clients and their caregivers (Focus 3).
- Adequately organize health and social services (prevention, treatment, rehabilitation, supportive care, and end-of-life care) (Focus 4).
- Prevent the onset of psychosocial, physical-health, and mental-health problems by reducing risk factors (Focus 6).
- Detect and take a proactive approach to physical-health problems (Focus 7).
- Detect and take a proactive approach to psychosocial and mental-health problems (Focus 8).
- Manage chronic conditions adequately (Focus 9).

To incorporate the highest possible number of standardized terms into the ICPs, we used the normative framework of the Ministry of Health and Social Services developed for the planning module of the RSIPA computerized solution (French acronym for Réseau de services intégrés pour personnes âgées [integrated service network for older people]) [69]. A normative framework is a reference document that supports the entry of standardized data into a computerized solution and their uses for informational purposes. RSIPA is a project involving the computerization of ISNs for the elderly population in our health- and social-services centers (www.sogique.qc.ca/Familles-de-services/Actifs-informationnels/RSIPA.aspx). We also used some terms from three internationally recognized classifications and nomenclatures: the International Classification of Functioning, Disability and Health (ICF) proposed by the World Health Organization (WHO) in 2001 [70]; the International Classification of Diseases, 10^th^ edition (ICD-10) [71]; and the International Classification of Nursing Practice (ICNP^®^) [72]. The ICF, which is a classification of health and health-related domains that uses standardized terms and framework to describe functions and disabilities as major components of health and describes body structures and functions, activity limitations, and participation restrictions. These domains are classified according to two lists: a list of body functions and structure, and a list of domains of activity and participation. Since an individual’s functioning and disability occur in a context, the ICF also includes a list of environmental factors, completing the ICD and going beyond mortality and disease. ICD-10 makes it possible to code diseases, trauma, and the reasons why people contact health services. The ICNP^®^ consists of a unified language system that proposes common terminology for diagnosis, action, and outcome assessment. In the case of the latter, we were especially interested in terms referring to actions. Much of this terminology can also be applied to other professions. Depending on availability and ICP development, additional classifications may certainly be used over the next few years. Note that having standardized terminology gives clinicians a common language that can improve their communication and reduce ambiguities. It also facilitates computerization and data extraction for a variety of purposes, in particular, assessing the quality of practices [73, 74].

### Literature review

This step involved gathering data on evidence-based results for the condition in question, as well as on the appropriate action to be taken. Clinical-practice guides were especially useful in this regard; observing what had been done in other health-care systems or organizations also proved valuable. The interdisciplinary task team examined the best scientific evidence in relation to the 29 SMAF items (e.g. mobility, sensory functions, incontinence, taking medication, etc.), social-function items from the Social SMAF (e.g. social network [75]), and the conditions most frequently found in older people (such as undernutrition, falling, depression, and social vulnerability). The review was carried out using the Medline, CINAHL, Ageline, Scopus, and Healthcom bibliographical databanks, as well as the government Web sites of various countries and organizations involved in developing good clinical practices, such as the National Institute for Health and Clinical Excellence (NICE) and the National Guidelines Clearinghouse. Lastly, we also consulted a number of documents and volumes featuring work done in a Quebec context [76].

The challenge in this step was to identify the most relevant and usable information. Accordingly, each member of the interdisciplinary task team individually reviewed the data collected with his/her clinical expertise and the problem to be identified or detected (e.g. documents on abuse drafted by social workers and case managers). Next, those data were shared with the other members and discussed at meetings. These discussions made it possible to identify contradictory opinions in order to reach a consensus when, for example, the literature available did not provide enough evidence to establish optimal practices. Lastly, all the information collected was organized according to generic interventions (i.e. that all clients might require) or interventions that addressed each Iso-SMAF autonomy profile specifically.

### Prevailing practice documentation

This step consisted in documenting prevailing practice on the basis of a review of files and other relevant documents in a health- and social-services center. An examination of the files of individuals aged 65 and over receiving home-care services was conducted by three members of the interdisciplinary task team. Using a standardized collection sheet, six clinical files per Iso-SMAF profile (i.e. a total of 84 files) were studied to document the most frequently used practices and resources and to determine if any had been omitted during preliminary ICP development. Another 30 files of people 65 and over admitted to the intensive functional rehabilitation unit, the geriatric short-term unit, and the day hospital (ten files per department) were examined to identify the aspects to be considered in enhancing service coordination.

## Results

**Figure 3:**
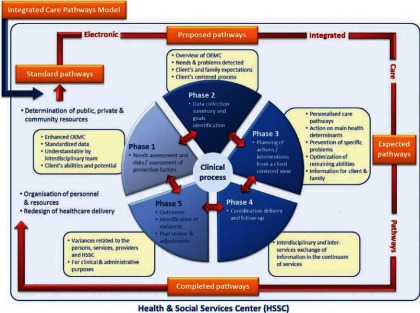
Clinical process.

ICPs can be represented by five levels of pathway production, which will be coupled to the five phases of a usual clinical process (see Figure 3). The pathway levels are named the model, standard, proposed, expected, and completed pathways. The **model pathway** takes in the reference ICPs developed in this study. The **standard pathway** is a translation of the model pathways for use in each home-care service of Quebec health- and social-services centers (HSSCs). It will take into account the specificity of each HSSC in term of public, private, and community resources in the continuum of their services. The **proposed**, **expected**, and **completed pathways** refer to the use of standard pathways for each home-care client.

The clinical process is part of the following phases: (1) needs assessment and assessment of risk/protection factors; (2) data-collection summary and goals identification; (3) planning of actions/interventions appropriate to the clinical situation and the goals and expectations of the client and his/her caregivers; (4) coordination, delivery, and follow-up; (5) identification of variances as well as review and adjustment of plans, as required. Note that each discipline and service may provide data inputs, such as their understanding of the situation or suggestions for improvement that may be required. So, the system will allow iterations and continual audits of the care of individual clients.

More specifically, the five phases can be described as follows:

### Needs assessment and assessment of risk/protection factors

The purpose of this phase is to document the source and causes of the needs of the client and his/her caregivers. Particular attention was also given to those essential elements that must be identified in order to reduce the incidence of frailty and disability. Since ICPs are highly dependent on the information generated by the assessment, it was necessary to analyze the OEMC content, which includes the following main fields: (1) health status; (2) lifestyle; (3) autonomy assessment; (4) psychosocial situation; (5) economic conditions; and (6) physical environment. Each field is divided into subsections (e.g. the ‘digestive function’ section is included in the ‘physical health’ field). For each subsection, the assessor must indicate by ‘yes’ or ‘no’ whether a problem has been identified. To aid in this clinical assessment, a checklist section incorporating certain data (e.g. nausea) is available for use in specifying and analyzing the situation. At present, 93 minimum standardized data sets are represented by these fields and subsections, and have been integrated into the RSIPA computerized solution. An examination of the OEMC, however, revealed that not all of the minimum essential standardized data to be highlighted during an assessment were available. For example, existing data are insufficient, and do not make it possible to systematically identify risk or protection factors for specific problems or detect physical-health, mental-health, and psychosocial problems. In particular, there is a lack of standardized data on personal and family health history, mental health, lifestyle, psychosocial situation, caregiver situation, economic conditions, and physical environment. The team’s work thus made it possible to identify which data were missing while respecting the existing OEMC structure. Certain principles were used in selecting those data. For example, the data had to give service providers a better comprehension of the client’s situation in the different areas assessed; be relevant to informed clinical decision making and assist in the care and service planning process; be clear and understandable to all service providers on the interdisciplinary team; and, when necessary, act as a care-quality indicator, a risk factor for a potential situation, a health-problem marker, or a marker suggesting that complementary assessment should be conducted. Nevertheless, the data had to represent only those situations most likely to be observed, not all possible situations. The objective is to support service providers and make them more effective, not constitute an additional burden. Lastly, the tool had to document the pertinent aspects related to the healthy aging conceptual model. Although, the OEMC synthetizes the information in describing needs and problems for each subsection, the ICP process comply with the philosophy of restorative care or care-reablement. These philosophies imply that we must focus on the client's abilities and potential for self-care and to perform everyday activities, not just on disabilities and available services [12, 47, 48].

### Summary of data collection and goals identification

This phase was aimed at establishing a concise depiction of the client’s situation in order to support the service provider’s clinical decision-making. To that end, several algorithms were constructed from the previous phase of assessment. Given the complex nature of the clinical situation involved in disability and the number of algorithms possible, we need a computer program that automatically generates the most relevant information. Thus, this phase received input from the **proposed** pathways. This information consists of the needs and problems detected; components of frailty; risks and protection factors identified (e.g. fall in the past year, undernutrition, social vulnerability); the functional and social autonomy evaluation summary (SMAF scores and Iso-SMAF profiles); the context in which various activities are carried out; the care and services provided by the different organizations as well as by the family; the expectations and specific desired outcomes of the client and his/her family; and, lastly, a description of the client’s environment. This consolidation, which the application presents in dropdown lists, helps service providers determine, in cooperation with the client, the latter’s priority needs and the goals to be reached in treatment-plan development and implementation. These goals could form a starting point for subsequent reviews to establish whether they were achieved.

### Planning of actions/interventions, and the delivery and follow-up phases

These two phases receive input from the production of ***proposed, expected,*** and ***completed*** pathways (see Figure 3). The ***proposed pathway*** included actions or interventions with timeframes suggested to the provider considering the clinical situation of his client and goals determined in the preceding phase. Standard or generic content (e.g. nutrition, abuse) is available for the pathways for all client types, in addition to disability profile specific content. Overall, this may involve prevention; teaching; consciousness-raising; coaching; guidance; counseling; maintaining autonomy; restoring autonomy, redesigning the environment; providing the family with support; conducting complementary assessments; referring the client to another professional, a public or private service, or a community organization; identifying possible sources of financial assistance; offering respite; or finding a new living arrangement; and ensuring follow-up. The ***expected pathway*** is produced in agreement with the client and illustrates actions or interventions for each individual client. If needed, service providers can also use a blank field to enter further specific action or add a goal using the list of suggested words. The ***completed pathway*** is that filled out and updated by the provider. Since standards for appointment and intervention frequency and related implementation deadlines are integrated into the planning process, ICPs may, for example, automatically issue alerts to inform users of a lack of action or a delay in treatment or consultation.

### Identification of variances; plan review and adjustment

In this fifth stage, both the degree to which goals have been reached and variances between the proposed, expected, and complete pathways are available, as is a list of the reasons underlying the variances as they relate to clients, service providers, and HSSC (e.g. client declining a service or service unavailable). These variances can also be aggregated for the purposes of administrative analysis, which is useful to service providers in readjusting the expected pathway, to the team in optimizing the process, and to managers in reengineering service organization.

## Discussion

This paper has described the development of integrated care pathways (ICPs) designed to meet the needs of frail and disabled older adults monitored by interdisciplinary teams from the local community integrated service networks. The main goal was to find a process that would help provide the right service to the right person accordingly to her profile of needs and expectations, while improving access, equity, and efficacy in a context of scarce resources.

The Institute of Medicine in the US underlines that evidence-based practice is a basic requirement for safe and effective clinical care [77]. To this end, ICPs are recognized as being useful for systemizing the evidence base [78]. Following an exhaustive inventory of the literature and a careful review of the available documentation, we found that no ICPs focused specifically on the complex autonomy-related needs. On the other hand, guidelines and a number of protocols for several specific conditions (e.g. nutrition, incontinence, falls, end-of-life care) do exist. Therefore, the task was to identify the most relevant and usable information and to attribute the evidence according to each disability profile. The biggest challenge was to select the right care that could be provided for whom and in which circumstances, considering the disability profile, risks factors, or some components of frailty of an individual. Another difficulty was also to take into account existing practices covering different services in the community and to translate evidence into the reality of our local structures. Issues such as the availability of services resulted sometimes in great discussions. We would like to make sure that these ICPs are not be too remote from day-to-day practice. Lastly, considering the quantity of elements considered in the production of ICPs, their development was very time-consuming and costly. In return, our ICPs focused on bringing a team together and enhancing communication and coordination. To achieve this goal, the roles of each discipline and communication channel were discussed and, most importantly, the members of the interdisciplinary task team adhered to the same evidence-based standards.

The Institute of Medicine also recommends that care processes need to be organized around client needs [77, 79]. The use of five levels in the production of pathways makes it possible to consider evidence-based practices and their adaptation to features of the organization but also the client-centered view. We hope that this approach will help tailor services to an individual’s circumstances and needs rather than planning services based on their availability.

We have to emphasize that these ICPs are a work in progress. Currently, the content of the preliminary version of the enhanced OEMC (paper version), as well as the ICPs developed to date, are submitted to provincial experts from various regions representing both rural and urban communities. For both the enhanced OEMC and the ICPs, the experts have to assess the appropriateness of the item or statement proposed for each disability profile or specific condition, as well as its clarity, using the Content Validity Index method [80]. They also have the chance to note comments or suggestions, which will then be dealt with by the multidisciplinary task group.

We are also currently working on integrating ICPs into a client computer system. The research team is working in partnership with designated analysts and programmers to reach a consensus on the final product so as to ensure that the ICPs provide support for the day-to-day practices of service providers while facilitating managers’ access to data useful in decision-making. This work involves the use of soft systems methodology (SSM). SSM is an approach that takes into account the social, political, and human aspects during the system development stage so that the focus is not solely on technological aspects. The approach tries to take into consideration the expectations of these interest groups and to have a better understanding of the system development context in order to reach a consensus on what an information system (IS) should be [81–83]. It provides the organization for clarifying the situation from *“What needs to be done”* to *“How are we going to do it”*. SSM divides itself into seven steps: (1) entering the problem situation; (2) expressing the problem situation; (3) formulating root definitions of relevant systems; (4) building conceptual models of human activity systems; (5) comparing the models with the real world; (6) defining changes that are desirable and feasible; and (7) taking action to improve the real-world situation. With a view to document the extent to which these stages are completed, follow-up will be provided by a research assistant with the help of committee-meeting qualitative observations and analyses.

Implementing these ICPs should generate many benefits. The individuals involved in this project believe that ICPs may help to reduce variations by identifying and acknowledging clients with similar needs while ensuring high quality and holistic care. The standardization of procedures and activities will allow service providers to better inform clients and their families on what to expect during the home-care episode, making for greater client participation and more informed decision making. With respect to both service providers and organizations, the knowledge of what must be planned, done, or assessed on the service continuum must improve time management, support decisions, promote better communication between professionals and partners, and result in a more efficient use of resources. Less-experienced staff members will also be able to participate fully in the treatment plan. Based on various data, HSSCs will be able to assess progress while observing intervention efficacy, and be better able to plan resources, organize work, and assess service quality.

Finally, considering that the Quebec health-care system tends to reduce cost by tightening eligibility requirements and restricting services to individuals with higher needs, one of our intentions was also to investigate alternative strategies in providing home services. The literature review convinced us that it was time to focus on prevention and on new approach of care (such as restorative care or re-ablement care) that promote independence instead of maintaining dependency. A restorative approach could be particularly well adapted for individuals with low to moderate levels of disability, although questions remain about which clients are likely to benefit more. Positive outcomes were reported recently in the literature, such as improved quality of life and functional status as well as reduced costs associated with a reduction in the ongoing use of home-care services after an intervention [49, 84]. In this way, providing timely interventions and education to encourage frail older adults to resume activity could bring our delivery of home-care services more closely in line with recent models of healthy aging. In this sense, the choice of the ‘healthy aging’ model was very helpful in guiding our work.
